# Design and Optimization of Multi-Stage TMR Sensors for Power Equipment AC/DC Leakage Current Detection

**DOI:** 10.3390/s23104749

**Published:** 2023-05-14

**Authors:** Xiaoxu Hu, Xuetao Duan, Wei Zhang, Yameng Fu, Yongfu Li, Pengcheng Zhao, Xudong Deng, Chuanxiang Yu, Jingang Wang

**Affiliations:** 1 State Key Laboratory of Power Transmission Equipment and System Security, Chongqing University, Chongqing 400044, China; 2 State Grid Chongqing Electric Power Research Institute, Chongqing 401123, China; 3 State Grid Chongqing Electric Power Company Ultra High Voltage Branch, Chongqing 400039, China

**Keywords:** tunnel magnetoresistance sensors, alternating current/direct current measurement, power equipment leakage current, size optimization

## Abstract

Tunnel magnetoresistance (TMR) can measure weak magnetic fields and has significant advantages for use in alternating current/direct current (AC/DC) leakage current sensors for power equipment; however, TMR current sensors are easily perturbed by external magnetic fields, and their measurement accuracy and measurement stability are limited in complex engineering application environments. To enhance the TMR sensor measurement performance, this paper proposes a new multi-stage TMR weak AC/DC sensor structure with high measurement sensitivity and anti-magnetic interference capability. The front-end magnetic measurement characteristics and interference immunity of the multi-stage TMR sensor are found to be closely related to the multi-stage ring size design via finite element simulation. The optimal size of the multipole magnetic ring is determined using an improved non-dominated ranking genetic algorithm (ACGWO-BP-NSGA-II) to derive the optimal sensor structure. Experimental results demonstrate that the newly designed multi-stage TMR current sensor has a measurement range of 60 mA, a fitting nonlinearity error of less than 1%, a measurement bandwidth of 0–80 kHz, a minimum AC measurement value of 85 μA and a minimum DC measurement value of 50 μA, as well as a strong external electromagnetic interference. The TMR sensor can effectively enhance measurement precision and stability in the presence of intense external electromagnetic interference.

## 1. Introduction

As the primary battleground for energy transformation, the power grid proposes to enhance its sensing and ubiquitous interconnection capabilities, actualize the state collection, real-time sensing, and online monitoring of power grid equipment, and construct a smart IoT system. Advanced sensing and measurement technologies are the foundation for real-time monitoring and fault prediction, and leakage current is one of the most crucial parameters for ensuring the stable and reliable operation of power systems and related application equipment, as well as power transmission safety [1,2,3]. Leakage current is the presentation of the results of complex operational conditions on power equipment, and its amplitude is very small, ranging from several hundred microamperes to several milliamperes. Currently, due to the increasing number of nonlinear load devices such as renewable energy and power electronics, the traditional AC system is evolving into a hybrid AC/DC power system, the leakage current signal is gradually approaching a complex waveform containing a variety of AC and DC components, and the traditional AC or DC sensors cannot achieve accurate measurement of the current, which is prone to causing protection switch misoperation, abnormal stator current, and other problems. Traditional AC or DC sensors are incapable of accurate current measurement, which can easily result in protection switch misoperation, anomalous state recognition, and misjudgment, thereby posing a significant threat to the safe operation of the power system [4,5,6]. Consequently, the design and development of high-precision and high-reliability AC and DC feeble current sensors is the current trend and focus of research in sensing technology.

The conventional current transformer is extensively used in the actual operation of power systems; however, it can only measure AC signals and has the disadvantages of a large size, a complex installation structure, a high price, and a narrow bandwidth [7]. Despite the Roche coil technology’s adequate bandwidth response, compact size, and low cost, it is primarily used for high current measurement and has limited capability for low frequency and low current measurement. In recent years, non-contact measurement technology has been rapidly developed in the context of smart grid sensing, where magnetic sensor technology is widely used for tiny current measurements due to its non-intrusive and electrically coupled isolation characteristics, allowing for high-accuracy measurements of currents below 100 μA [8,9,10,11]. Hall effect sensors are currently the most prevalent non-contact sensors on the market [12,13,14] due to their device simplicity, integrated processability, and low cost. However, silicon-based Hall sensors are extremely temperature sensitive, resulting in large output deviations, low measurement accuracy, and susceptibility to voltage breakdown, which cannot meet the demand for digital and highly dependable power system AC/DC leakage current measurement technology in the new era of the smart grid. Fluxgate current sensors are more sensitive to external magnetic fields and have less temperature drift and greater sensitivity than Hall current sensors [15,16], making them suitable for measuring small signals. Yang et al. [17] enhances the bidirectional saturated fluxgate principle and proposed a new detection method with improved temperature stability and linearity in small current measurements. The literature [18] proposed a controlled greedy pairing strategy to minimize the zero offset of the fluxgate sensor from 131.27 μA to 19.5 μA to enhance the measurement accuracy of the fluxgate sensor. Although the aforementioned study proposes enhancements to further enhance the adaptability of fluxgate sensors for feeble small current measurements, it also increases the cost, size, and complexity of the sensor structure and the measurement principle.

Magnetoresistive sensors are capable of detecting magnetic field domains of 10^−9^–10^−2^ T [19], which has substantial advantages for measuring microampere currents [20,21,22]. The majority of the newly developed magnetoresistive sensors are anisotropic magnetoresistive (AMR) [23], giant magnetoresistive (GMR) [24], and tunneling magnetoresistive (TMR) [25] sensors. Compared to AMR and GMR, the magnetoresistance ratio of TMR can reach more than 200% [26], which has more outstanding measurement performance advantages, and it has the characteristics of high sensitivity, low cost, low power consumption, and small size, without complex excitation circuit, and is anticipated to be manufactured as an advanced intelligent sensor device for weak current measurement [27]. Currently, non-contact measurement forms are required for the field measurement of leakage current of power equipment, and the complex working conditions of the field make it difficult to improve the accuracy of weak current measurement with magnetoresistive sensors due to the influence of stray magnetic interference. Magnetoresistive current sensors are primarily available in open-loop and closed-loop configurations. For open-loop structures, there are typically two types: non-magnetic rings and magnetic rings. The non-magnetic ring structure sensor deploys the magnetoresistive sensing device directly around the wire, is simple to install, and is available in several configurations. The literature [28] introduced a U-shaped magnetic shielding structure to the GMR sensor to shield the external stray magnetic field in the X-axis direction; however, it did not improve the front-end magnetic field measurement sensitivity, and its experimental validation for 20 A high current measurement did not include the effect of microampere level measurement performance. Xu et al. [29] employ a magnetless ring sensor structure with four TMR chips to eliminate the influence of stray magnetic fields and increase the precision of DC and AC measurements. Nevertheless, the magnetless ring structure is generally suited for high-current detection in power systems and has limited resistance to magnetic interference and amplification for high-precision detection of feeble currents as low as mA or even μA. The open-loop structure with a magnetic ring places the magnetoresistive sensing chip in the open gap of the magnetic ring, which has stronger advantages than the non-magnetic ring structure for shielding external magnetic field interference, amplifying the front-end magnetoresistive sensing chip magnetic field measurement size, and providing electrical isolation to a certain extent, which has greater safety in engineering practice. The non-contact weak current testing method based on giant magnetoresistive sensors described in the literature [30] can accomplish current testing at the tens of mA level with a bandwidth capable of reaching MHz, but cannot measure microampere currents. Lei et al. [31] employed an open-loop type with magnetic ring TMR current sensor structure and introduced temperature compensation, resulting in a maximum measurement error of 0.46% in the measurement range of −200 mA to 200 mA; however, the performance situation for 0–1 mA microampere level measurements is unclear. Hu et al. [32] developed a contactless microcurrent sensor using the tunneling magnetoresistive effect and a low-noise design for microcurrent sensors with a minimum current amplitude of 280 μA. Nevertheless, in the actual operation of some power equipment, leakage current measurements are as low as 100 μA and the magnitude of the resulting magnetic characteristic quantity is in some cases below the 0.01 μT level. The closed-loop sensor is based on an open-loop structure with a magnetic loop, and the feedback current obtained on the output side is introduced through a feedback resistor into the magnetic loop at the source to generate a feedback magnetic field. In the literature [33], a TMR-based magnetically balanced weak sensor was designed, and simulation experiments confirmed a 0.7% measurement error when measuring 1–10 mA current. However, the closed-loop structure increases the design cost and intricacy of the sensor, whereas the introduction of the negative feedback link tends to cause errors in the closed-loop loop, which are not more significant in terms of their applicability for weak current measurements.

This paper proposes a TMR AC/DC leakage current sensor with a multi-stage magnetic ring structure to improve the measurement sensitivity and anti-magnetic interference capability of TMR current sensors based on the open-loop design concept. Specifically, it can increase the accuracy and stability of the TMR current sensor for small currents without increasing the sensor size or processing complexity of the circuit. The experimental test and analysis results demonstrate that the proposed multi-stage TMR current sensor can enhance the accuracy and interference resistance of feeble current measurement. The primary characteristics of the structure and design scheme of the multi-stage TMR leakage current sensor are as follows:

- The method possesses excellent front-end magnetic field measurement sensitivity and anti-magnetic interference performance;
- The method can increase the accuracy and stability of microampere level current sensor measurements without increasing the size of the sensor or the processing complexity of the circuit;
- An improved non-dominated ranking genetic algorithm (ACGWO-BP-NSGA-II) is proposed for nonlinear mapping to assure the optimal performance of the front-end magnetic measurement of the sensor with the smallest possible size.

The structure of this paper is as follows: In Section 2, the design principles and concepts of the multi-stage TMR sensor are introduced, and then the front-end magnetic characteristics of the sensor are analyzed by the finite element method. In Section 3, the multi-objective optimization concept of the multi-stage magnetic ring structure, the ACGWO-BP-NSGA-II multi-objective optimization method, and the analysis of the optimization results are introduced. Section 4 describes the experimental testing and analysis of the sensor in detail. The last section of this paper is Section 5.

## 2. Multi-Stage Open-Loop TMR Sensor Characterization

### 2.1. Multi-Stage Open-Loop TMR Sensor Structure

**Figure 1:**
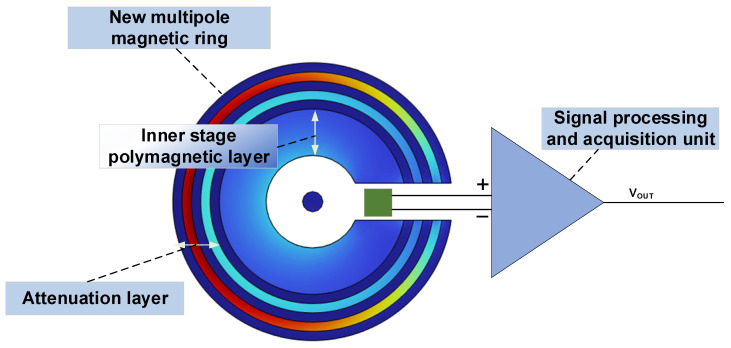
Multi-stage open-loop TMR sensor structure diagram.

Figure 1 depicts the multi-stage open-loop TMR sensor structure proposed in this paper. The multi-stage TMR sensor is composed of a multi-stage magnetic ring at the front end, TMR sensing module, and a signal processing and acquisition module, which converts the measured current signal into a voltage signal output. The multi-stage open-loop TMR weak current sensor’s design advantage is a new multi-stage magnetic ring structure with high magnetic field stability. It is composed of alternating combinations of high magnetic permeability and high conductivity media, and the overall structure can be divided into two main parts based on the functional distinction: the inner stage pole magnetic layer and the attenuation layer.

Under a closed-loop magnetic circuit, various magnetic circuit media can be viewed as a series magnetic circuit, whereas adjacent media can be analyzed as parallel magnetic circuits. As shown in Equation (1), based on Ohm’s law of magnetic circuits, magnetic resistance *R_m_* is proportional to magnetic circuit length *l* and inversely proportional to magnetic circuit cross-sectional area *S* and magnetic permeability *μ*.

(1) $$R_{m}=\frac{l}{\left(\mu S\right)}$$

For the inner stage polemagnetic layer, the high permeability magnetic ring provides a magnetic flux path *R_m_*_1_ with low reluctance, and the adjacent highly conductive material’s parallel reluctance *R_m_*_2_ and air reluctance *R_ma_*_1_ are both several thousand times greater than *R_m_*_1_. The guide wire traverses the center of the magnetic ring, and the ring-shaped dispersed magnetic field generated by the target current will be accumulated within the inner magnetic ring. Referring to relevant literature studies [34,35], it is known that the magnetic field generated by the target current is accumulated and amplified in the air gap of the magnetic ring and is proportional to the magnitude of the current in conjunction with the Biot–Savard law. The TMR induction module is positioned in the center of the inner stage of the aggregated magnetic layer gap. Using the tunneling magnetoresistance effect mechanism and the Wheatstone bridge structure, the output of the voltage signal proportional to the current signal is achieved. The generated electrical signal is amplified in the subsequent circuit, and the acquisition and measurement of the AC/DC current are completed at the output. From the preceding analysis, it is also possible to conclude that the front-end magnetic field sensitivity of the multi-stage TMR current sensor is primarily related to the structural properties of the inner stage polymagnetic layer.

The attenuation layer consists of a highly conductive material alternating with a high permeability material on the outside of the inner stage polymagnetic layer. The high conductive layer can produce an eddy current effect to weaken the external interference magnetic field and repel the interference magnetic field outside the magnetic path generated by the target current, thereby achieving eddy current elimination of the alternating magnetic field resulting from high-frequency magnetic interference. The low reluctance magnetic flux path formed by the high permeability material directs the magnetic induction lines of external interference to pass along the wall of the high permeability layer, thereby realizing the flux shunt of external quasi-static interference. According to Equation (2), the external electromagnetic propagation coefficient *τ* is determined. Any combination of dielectric constant *ε*, permeability *μ*, and conductivity *σ* that increases the attenuation constant *α* is capable of suppressing the interference from an external magnetic field. The disparity between the number of attenuation layer dielectric combination levels and the various diameters results in distinct dielectric combination strategies. When a suitable combination form is employed, the attenuation layer can not only reduce the external leakage of the magnetic field generated by the target current, but can also enhance the signal-to-noise ratio of the magnetic field measurement induced by the weak current.

(2) $$\tau =\sqrt{j\omega \mu \left(\sigma +j\omega \varepsilon \right)}=\alpha +j\beta$$

In Equation (2), *β* is the phase constant.

The aforementioned theoretical foundation serves as the foundation for the design of multi-stage TMR feeble current sensors.

**Figure 2:**
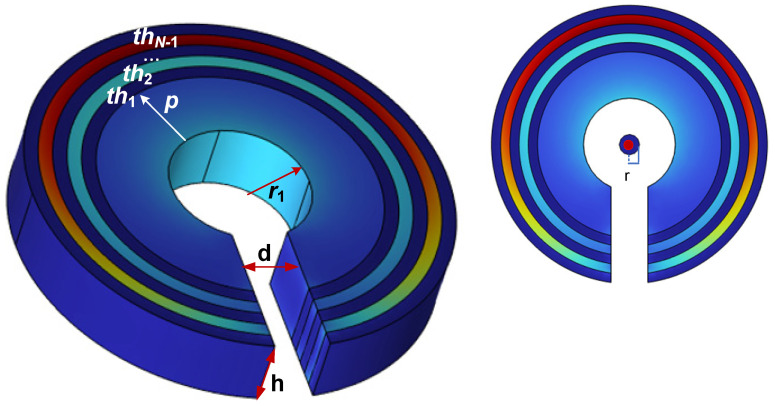
Simulation model of multi-stage TMR sensor front-end.

In Figure 2, the thickness of the inner stage polymagnetic layer is denoted by *p*, *th*_1_-*th_N_*_1_ denotes the thickness of the attenuation layer at all levels, *h* denotes the multi-stage ring height, *d* denotes the multi-stage ring air gap width, *r*_1_ denotes the inner radius, *r* denotes the wire radius and *N* represents the total number of stages of the multi stage magnetic ring. As the weak current measurement scenario has low requirements for antimagnetic saturation capability, the high magnetic permeability material is selected from permalloy, which maintains high permeability in weak magnetic fields, and the high conductive material is selected from aluminum with good eddy current elimination performance in AC magnetic fields [36]. The leakage current of power equipment is typically less than 100 mA, so a 2 mm copper wire radius is sufficient to satisfy the current flow specifications. The material and size parameters for the model are shown in Table 1 and Table 2.

To evaluate the effectiveness of the new multi-stage magnetic ring structure in terms of its ability to gather magnetism and resistance to external magnetic interference, and to facilitate the comparison of the performance of the conventional single-ring structure, the magnetic field measurement sensitivity *A* and the relative error *MC* of magnetic field measurement under the influence of interference currents are defined to describe the measurement performance of the sensor induction front-end for weak current measurement scenarios.

(3) $$A=\frac{\left|B_{mr}\right|}{\left|B_{0}\right|}$$

where *B*_0_ is the magnetic induction measured by the TMR sensor under a single wire, and *B_mr_* is the magnetic induction measured by the TMR sensor under a magnetic ring structure.

(4) $$MC=\frac{\left|B_{mi}-B_{nmi}\right|}{\left|B_{nmi}\right|}\times 100\%$$

where *B_nmi_* is the magnetic induction measured by the TMR sensor without interference, *B_mi_* is the magnetic induction measured by the TMR sensor with interference, and the smaller the *MC* means the higher the accuracy of the magnetic field measured by the TMR.

### 2.2. Magnetic Sensitivity Analysis

The current *I* is applied through the wire, and a parametric scan is set from 1 mA to 10 mA with a step of 1 mA to simulate the small current measurement, and a steady-state solver is set to change multi-stage magnetic ring series *N* = 1–6 to obtain the magnetic field distribution of current-dependent magnetic induction at the TMR deployment point at all levels, and a single wire model is added to obtain the magnetic induction at the same location for comparison and analysis, where *N* = 1 is the special case of a single magnetic ring structure.

**Figure 3:**
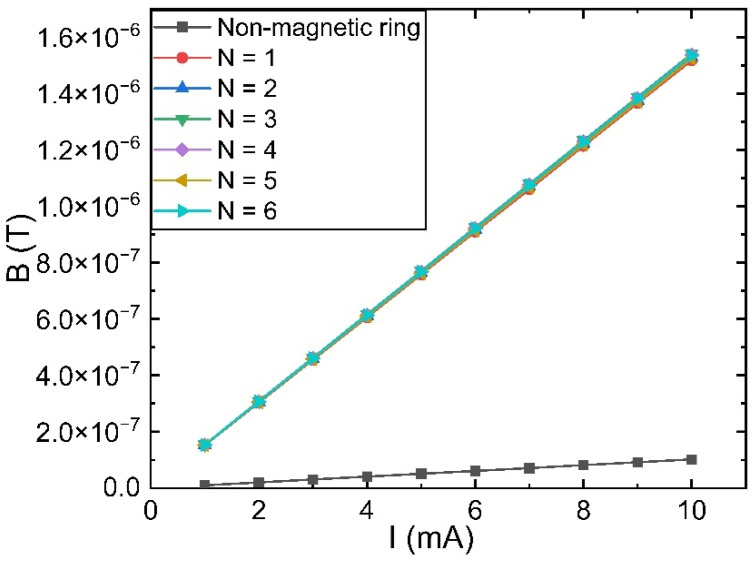
Variation of magnetic field magnitude with current at the measurement point.

**Figure 4:**
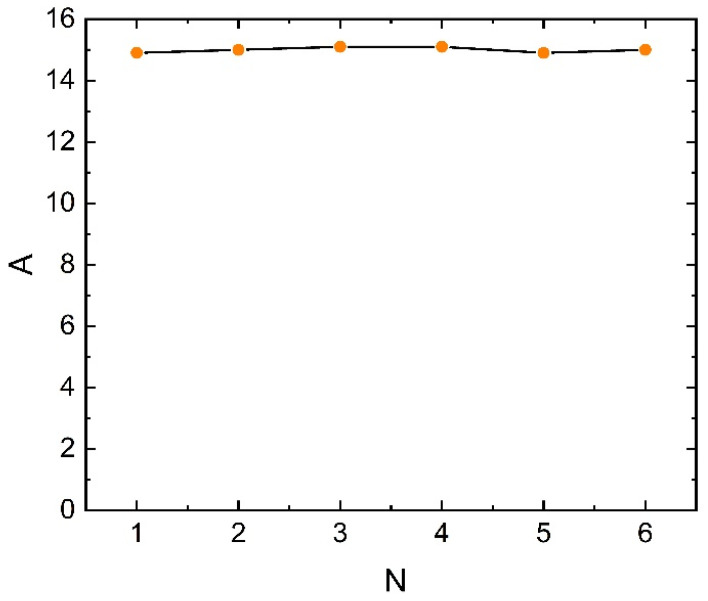
Sensitivity of the magnetic field measurement at the measurement point as the current changes.

As depicted in Figure 3, the total magnetic induction intensity and magnetic induction intensity in each direction at the TMR measurement point increase linearly with the change in current under the multi-stage magnetic ring structure, which can significantly enhance the magnetic field measurement sensitivity and facilitate the magnetic field measurement and current inversion calculation. Comparing the calculated values of magnetic field sensitivity at each level, as depicted in Figure 4, reveals that the amplification capability of the multi-stage ring structure is primarily dependent on the thickness of the inner layer, whereas the thickness of the attenuation layer has no impact on the amplification capability.

**Figure 5:**
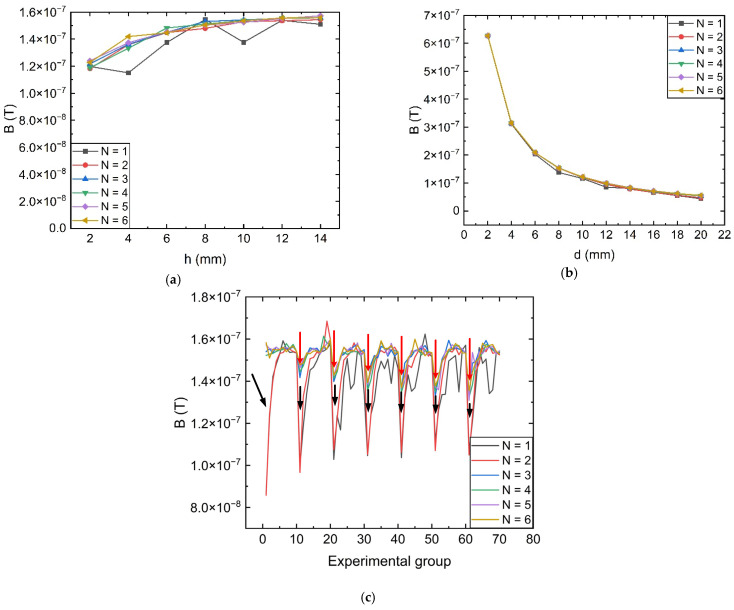
Magnetic field characteristics at the measurement point under the change of the new multi-stage size structure: (**a**) The magnitude of the magnetic field at the air gap as *h* varies; (**b**) The magnitude of the magnetic field at the air gap as d varies; (**c**) The magnitude of the magnetic field at the measurement point that varies synchronously with *r*_1_ and *p*.

The magnetic field amplification capacity at the air gap of the single-ring structure is closely related to its dimensional structure, so the effects of varying the ring height *h*, the open air gap *d*, the inner radius *r*_1,_ and the ring thickness *p* on the sensitivity of magnetic field measurement in the air gap are obtained in Figure 5. Among them, the experimental set depicted in Figure 5 crepresents the combination of experimental dimensions ranging from 3 mm to 15 mm for *r*_1_ and 1 to 20 mm for *p*.

From the above simulation results, it can be seen that the magnetic field measurement size can reach a more stable value when *h* is greater than 8 mm, while the opening gap width *d* and the size of the inner and outer radius of the magnetic ring are set to affect the performance of the front-end magnetic field measurement of the sensor. The magnetic field amplification is inversely proportional to the opening gap width *d*. The rate of decline is fast when the opening gap is small, and the magnetic field strength measured by the sensor is at a lower level and declines slowly as *d* expands further. Therefore, the design of the opening air gap width *d* should be as small as possible to ensure that the TMR sensor chip is put into the premise.

The black arrows in Figure 5c indicate depressions with *p*-value thicknesses of 1–8 mm, while the red arrows indicate depressions with *p*-value thicknesses of 1–3 mm. The simulation results show that if a single magnetic ring structure is used, the ring thickness p must be greater than 8 mm to achieve a higher magnetic field gathering capability at the air gap, whereas a magnetic ring structure with three or more stages and a single stage with a gathering layer thickness p greater than or equal to 4 mm can achieve the same result. At the measurement point, the magnetic field measurement sensitivity can be high, yielding improved measurement results overall for the magnetic field measurement value. Similarly, when the magnetic ring structure has good magnetization capability, the magnetic field measured at the same measurement point has a small range of variation with the increase in the inner radius, indicating that the inner diameter setting has less of an effect on the sensitivity characteristics of the magnetic field measurement at the deployment point.

### 2.3. Analysis of Anti-Magnetic Capacity

**Figure 6:**
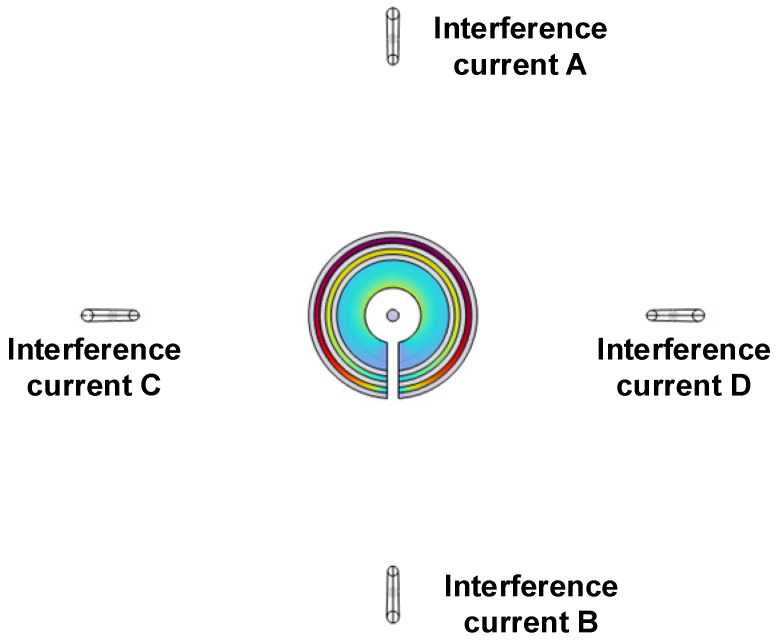
Interference position placement diagram.

**Figure 7:**
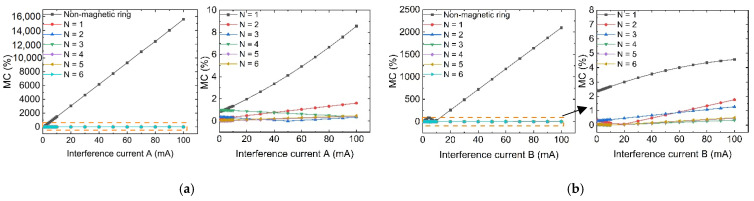

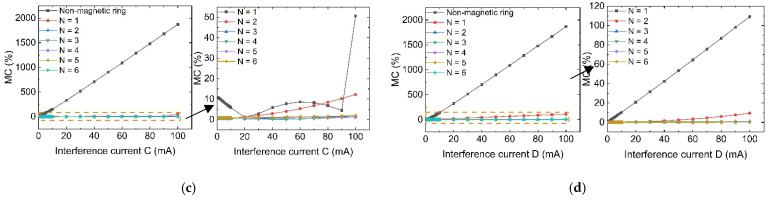
Schematic diagram of the TMR front-end anti-magnetic interference performance with and without the magnetic ring structure at different angles of interference: (**a**) Under Interference A; (**b**) Under Interference B; (**c**) Under Interference C; (**d**) Under Interference D.

To simulate magnetic field interference from different angles, the interference current wires are placed around the sensor at 90-degree intervals, taking into account the symmetry of the spatial structure. Four interference current sources are set as shown in Figure 6, the interference currents are labeled with the letters A–D, and *I*′_A_–*I*′_D_ is used in the text to indicate that the simulation sets the distance *L* = 0.1 m from the interference currents. The parameters correspond to the default values. In the simulation setup, the external interference size is gradually increased to obtain the relative error results of TMR magnetic field measurement under different structures as shown in Figure 7.

As can be seen from Figure 7, the multi-stage magnetic ring structure proposed in this paper can significantly improve the anti-magnetic capability of sensor measurements. Under the single magnetic ring structure, the *MC* is as high as 109% when *I*′_D_ = 100 mA interference, which indicates that the single magnetic ring structure is still weak for magnetic field interference at all angles. Additionally, under the action of *I*′_D_ = 100 mA, the *MC* of the 2-stage magnetic ring structure is reduced to 10%, while the anti-magnetic interference ability of the sensor is further improved with the increase in *N*. The relative error size changes smoothly. However, the increase in the number of stages does not bring a sustained error reduction, *N* = 3–6, and the sensor magnetic field measurement error is similar, indicating that the three-stage magnetic ring structure can already achieve a better anti-magnetic capability.

**Figure 8:**
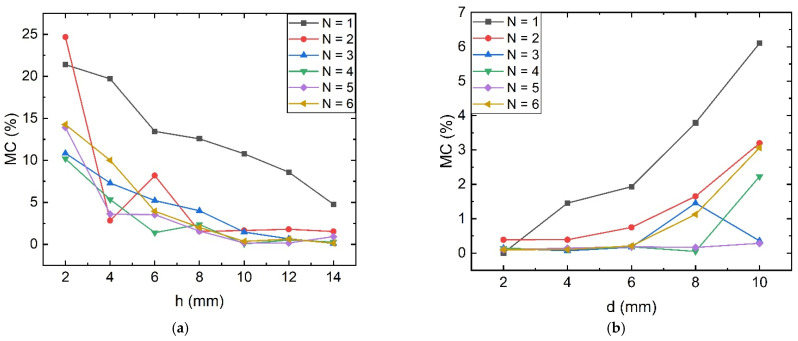
Effect of height h and opening gap d on magnetic interference immunity of TMR front-end: (**a**) With the variation of height *h*; (**b**) With the variation of the opening slit *d*.

To further investigate the effect of multi-stage magnetic ring structure on the anti-magnetic interference performance of multi-stage TMR current sensor front-end, the ring height h and opening gap width *d* are varied to acquire the simulation calculation results depicted in Figure 8.

As shown in Figure 8, when keeping the model set as the initial size and changing only the height h and air gap width *d*, under the action of *I*′_B_ = 100 mA, it can be seen from the simulation results that the overall trend of the anti-magnetic interference capability of the multi-stage magnetic ring decreases and increases with the increase in *h* and *d*, respectively. After *d* is less than 6 mm and *h* is greater than 10 mm, the anti-magnetic capability can be maintained at a stable low level.

For different combinations of attenuation layer thicknesses, the antimagnetic capability also shows different characteristics. Fix the size, keep the magnetic ring *r*_1_ = 10 mm, *r*_1_ = 10 mm, *p* + $\overset{N-1}{\underset{i}{\sum }}th_{i}$ = 10 mm unchanged, take the number of levels *N* as 1–6, set *I* = 1 mA, *I*′ = 100 mA, and set the thickness of each stage as shown in Table 3.

**Figure 9:**
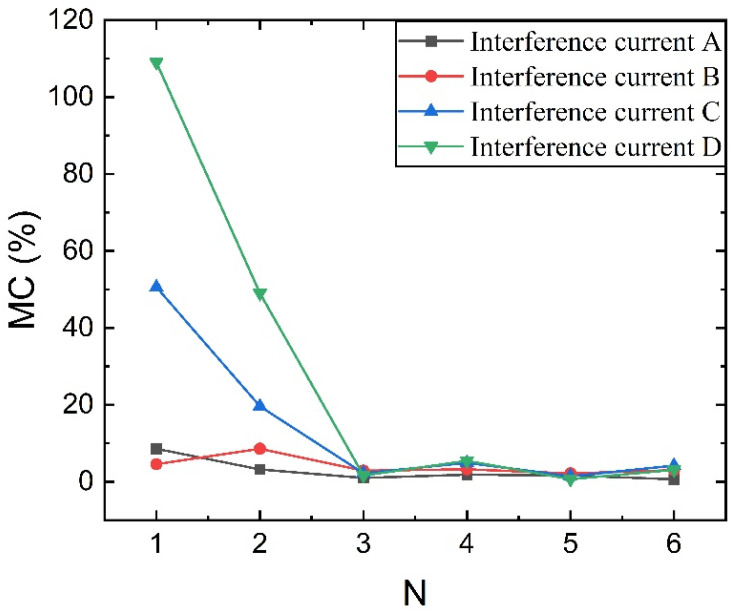
Analysis of TMR front-end anti-magnetic interference performance with the same ring thickness and different N-stage settings.

As shown in Figure 9, when *N* ≥ 3, the magnetic field measurement accuracy of multi-stage polymagnetic ring structures with the same ring thickness is significantly lower than that of *N* = 1 (single-stage polymagnetic layer structures), demonstrating once again the effectiveness of the proposed structure’s anti-magnetic interference capability.

Comprehensive analysis of Section 2.2 and Section 2.3 reveals that the matching effect of the magnetic ring size has a significant impact on the front-end magnetic field measurement sensitivity and anti-magnetic interference capability performance of the sensor. Taking the right size combination can further be able to suppress the leakage of the target magnetic circuit and improve the signal-to-noise ratio of the magnetic field measurement of weak current induction so that the front-end magnetic field measurement sensitivity is increased. It is necessary to optimize the overall measurement precision of the multi-stage TMR sensor for feeble AC/DC signals.

## 3. Dimensional Optimization Design of ACGWO-BP-NSGA-II

### 3.1. Optimization Target Establishment

The objective of the multi-stage magnetic ring structure is to ensure that the largest possible target current-generated magnetic field is measured at the TMR deployment measurement point, as well as to improve the antimagnetic capability of the sensor front end; therefore, the objective of the structure optimization is to improve the deployment measurement point *B* and reduce the relative magnetic field error value *MC* as much as possible, while meeting the miniaturization requirements. Since the relationship between the magnetic ring size structure and the solution target is a nonlinear and complex solution problem in the spatial magnetic field calculation process, too many optimization parameters will result in overly complex, time-consuming, and constraining conditions for the establishment of the optimal objective function relationship, which must be screened for parameter optimization.

In this paper, a multi-stage TMR sensor with an inner diameter *Φ* of 20 mm and an open air gap right into the TMR sensing module is designed and set at *d* = 8 mm. Combining the analysis in Section 2.2 and Section 2.3, it can be seen that when *h* is greater than 8 mm, the influence on the magnetic sensitivity and anti-magnetic interference ability is small, while when the number of stages *N* ≥ 3 increases with the increase in *N,* the increase in anti-magnetic capability is not very obvious, and the 3-stage magnetic ring structure is often consistent with the 5-stage or even 6-stage magnetic ring structure in terms of magnetic field sensitivity characteristics and anti-magnetic capability. In order to reduce the production cost and optimize the overall volume of the sensor, *h* = 10 mm and the number of stages *N* = 3. Considering the placement size of the TMR module, the thickness of the inner stage of the polymagnetic layer *p* needs to be larger than 6 mm, and it can be seen from the figure that when *p* is larger than 6 mm, the magnetic field measurement sensitivity under the three-stage magnetic ring structure is almost the same and almost at a stable value, which can ensure the magnetic field measurement sensitivity at the TMR deployment point.

Based on the above analysis, the thickness *p* of the inner stage polymagnetic layer and the thickness *th*_1_–*th*_2_ of the attenuation layer in the multi-stage magnetic ring structure are finally determined as the structure dimensional optimization parameters, and the design is expressed as a comprehensive problem with a dual objective optimization as shown in Equation (5):

(5) $$\begin{array}{c} \min M=\overset{4}{\underset{j=1}{\sum }}z_{j}MC_{j}\left(I^{\prime },p,th_{1},th_{2}\right)\\ \min C=p+th_{1}+th_{2}\\ st.\left\{\begin{array}{c} p\in \left[PL,PH\right]\\ th_{1}\in \left[HL,HH\right]\\ th_{2}\in \left[TL,TH\right] \end{array}\right. \end{array}$$

where *M* is the combined error of magnetic field measurement; *C* is the total thickness of the magnetic ring; *MC_j_* represents the true mapping relationship of the magnetic field measurement error resulting from the jth angle interference at a predetermined interference intensity; *z_j_* represents the weight size of magnetic field interference at each angle; *z*_1_–*z*_4_ represent the influence weights of *I*′_A_–*I*′_D_, which are determined based on the information and fluctuation of the relative *MC* size obtained at each angle; *PL* is the minimum value of the cohesive magnetic layer thickness, whereas *PH* is the utmost value. *HL* is the minimum value of the secondary aluminum layer, while *HH* is the maximum value of the secondary aluminum layer. *TL* is the minimum value of the tertiary magnetic layer, while *TH* is the maximum value.

### 3.2. ACGWO-BP-NSGA-II Optimization Process

The characterization *MC_j_* fitting mapping *F* for the optimization problem in this study must first be identified. To generate the fitted samples using COMSOL Multiphysics software, the mapping input parameter ***X*** of M can be represented as ***X*** = {*x_i_*} = {*I_i_*′, *p_i_*, *th*_1*i*,_ *th*_2*i*_}. This first refers to the model-building procedures in Section 2. The following are the precise steps in sample generation:

(1) Build the multi-stage magnetic ring structure model. Determine the model dimensions *r*_1_ = 10 mm, *d* = 4 mm, *h* = 10 mm, the number of stages *N* = 3, initialize *p* = *th*_1_ = *th*_2_ = *th*_3_ = 1 mm, and perform relevant material and boundary settings and meshing.
(2) Set the scan range of optimized dimension parameters. Set the scan range of the first stage size *p* to 6–15 mm; the variation range of *th*_1_ and *th*_2_ to 1–10 mm.
(3) Solve the calculation. Set the interference current *I*′ to 1–1000 mA, and change *p*, *th*_1_, and *th*_2_ to obtain the magnetic field measurement error *MC*_1_(***X***)–*MC*_4_(***X***) obtained for sample ***X*** under four interferences according to the setting in Equation (2), then the fitted sample for mapping *MC_j_*() is (***X***, *MC_j_*(*X*)).

After obtaining the fitted samples, the mapping relationship is fitted and calculated. In this paper, the goodness-of-fit *R*^2^ and the mean absolute error *MAE* are chosen to evaluate the fit.

(6) $$R^{2}=1-\frac{\sum_{i=1}^{n}{\left(y_{i}-{\hat{y}}_{i}\right)}^{2}}{\sum_{i=1}^{n}{\left(y_{i}-\frac{\sum_{i=1}^{n}y_{i}}{n}\right)}^{2}}$$

(7) $$MAE=\frac{1}{n}\times \overset{n}{\underset{i=1}{\sum }}\left|y_{i}-{\hat{y}}_{i}\right|$$

where *y_i_* denotes the true value of the sample, $\hat{y_{i}}$ denotes the fitted value of the ith sample, and *n* is the number of samples. The larger the *R*^2^, the better the fit and the optimal value is 1; the smaller the *MAE*, the smaller the relative error between the fitted value and the true value.

#### 3.2.1. ACWGO-BP Nonlinear Fitting Algorithm

The characteristic relationship between *MC_j_* and *I*′, *p*, *th*_1_, and *th*_2_ parameters for each angle now exhibits a strong nonlinearity with a large value span interval, and the emergence of neural networks has facilitated a novel approach to solving this issue. BP neural networks have strong self-learning and self-adaptive capabilities and have been effectively applied to nonlinear fitting solution problems. Nonetheless, the BP algorithm is based on a gradient or Newton-type deterministic algorithm, which makes it sensitive to initial conditions and prone to random approximation and local optimum. Therefore, this paper proposes to train the weights and threshold parameters of the BP network using an enhanced version of the gray wolf algorithm in order to acquire the optimal parameters of the BP neural network and the global optimal solution for the nonlinear fitting solution to *MC_j_*.

The solution idea of the gray wolf algorithm mimics the social leadership and hunting behavior of gray wolf society, and divides the population individuals into four categories: head wolf (*χ*_1_), gray wolf (*χ*_2_), gray wolf (*χ*_3_), and other gray wolf individuals (*χ*_3_). The traditional GWO optimization algorithm mainly includes three steps: roundup, pursuit, and attack [37].

(8) $$\vec{U}\left(t+1\right)=\vec{U_{p}}\left(t\right)-\vec{AA}\cdot \left|\vec{CC}\times \vec{U_{p}}\left(t\right)-\vec{U}\left(t\right)\right|$$

(9) $$\vec{AA}=2\vec{a}\times \vec{rr_{1}}-\vec{a}$$

(10) $$\vec{CC}=2\times \vec{rr_{2}}$$

where $\vec{U}$ is the position vector of the gray wolf, *t* is the current iteration number, $\vec{U_{p}}$ is the prey position vector, $\vec{AA}$ and $\vec{CC}$ are the coefficient vectors, $\vec{rr_{1}}$ and $\vec{rr_{2}}$ are the random vectors between [0, 1], and a is the linear decreasing convergence coefficient from 2 to 0, as shown in Equation (11).

(11) $$\vec{a}(t)=2-\frac{2t}{\;t_{\max}}$$

where *t*_max_ is the total number of iterations.

When the gray wolf group finds the hunting target, the positions of *χ*_1_, *χ*_2_ and *χ*_3_ are updated in the search space using Equations (12)–(14), and the optimal solution of the desired problem is obtained under the current iteration according to Equation (15).

(12) $$\vec{U_{1}}\left(t\right)=\vec{U_{\chi_{1}}}\left(t\right)-\vec{AA_{1}}\times \left|\vec{CC_{1}}\times \vec{U_{\chi_{1}}}\left(t\right)-\vec{U}\left(t\right)\right|$$

(13) $$\vec{U_{2}}\left(t\right)=\vec{U_{\chi_{2}}}\left(t\right)-\vec{AA_{2}}\times \left|\vec{CC_{2}}\times \vec{U_{\chi_{2}}}\left(t\right)-\vec{U}\left(t\right)\right|$$

(14) $$\vec{U_{3}}\left(t\right)=\vec{U_{\chi_{3}}}\left(t\right)-\vec{AA_{3}}\times \left|\vec{CC_{3}}\times \vec{U_{\chi_{3}}}\left(t\right)-\vec{U}\left(t\right)\right|$$

(15) $$\vec{U}\left(t+1\right)=\frac{\vec{U_{1}}\left(t\right)+\vec{U_{2}}\left(t\right)+\vec{U_{3}}\left(t\right)}{3}$$

The GWO algorithm, despite its better convergence speed, still searches for the global optimal solution, is the influence of the search path, and falls into local optimality. To enhance the GWO exploration capability, the GWO algorithm is improved to obtain the Adaptive Gray Wolf Optimization Seeking Algorithm (ACGWO). The specific improvement points are as follows:

(a) Chaos mapping initialization. Tent chaos mapping is introduced in the initialization to ensure the uniformity of the initial population distribution and the diversity of the populations, and to speed up the convergence of the GWO algorithm in the exploration space.The sequence of Tent chaos mapping $Z\left(t+1\right)$ is shown in Equation (16)
  (16) $$Z\left(t+1\right)=\left\{\begin{array}{ll} \begin{array}{c} \frac{Z\left(t\right)}{u}\\ \frac{1-Z\left(t\right)}{1-u} \end{array} & \begin{array}{c} 0\leq Z\left(t\right)\leq u\\ u\leq Z\left(t\right)\leq 1 \end{array} \end{array}\right.$$
  where *u* is a random number between [0, 1].
(b) Then, the initial position sequence of gray wolf is is shown in Equation (17).
  (17) $$\vec{U}\left(t\right)={\vec{U}}_{\min}\left(t\right)+Z\left(t\right)\times \left({\vec{U}}_{\max}\left(t\right)-{\vec{U}}_{\min}\left(t\right)\right)$$
(c) Adaptive descent strategy. Since the path of GWO in the process of finding the optimal solution, exploring the optimal search itself is not linearly convergent. The traditional convergence path presents linear convergence as shown in Equation (11), and cannot reflect the actual optimization approach and release the inherent ability of GWO’s superior search. The adaptive descent strategy as shown in Equation (18) is introduced to ensure its balance in local and global search.
  (18) $$\begin{array}{cc} \vec{a}\left(t\right)=2\times \left(1-\frac{n-1}{pp-1}\pm \kappa \right) & \frac{n-1}{pp}\leq t\leq \frac{nt_{\max}}{pp} \end{array}$$
  where *pp* is the adaptive step size, *n* = 1, 2, …, *pp*, *κ* is the random compensation factor [0, 0.1]. when *n* takes 1 and *pp*, the random compensation factor *κ* takes 0.
(d) Location update correction. Finally, inspired by the literature [38], a similar correction was made to the location update formula to play and use individual information to guide the individual search capabilities of wolves.
  (19) $$\vec{U}\left(t+1\right)=bb_{1}\times cc_{1}\times \frac{{\vec{U}}_{1}\left(t\right)+{\vec{U}}_{2}\left(t\right)+{\vec{U}}_{3}\left(t\right)}{3}+bb_{2}\times cc_{2}\times \left(\vec{CC_{1}}\times \vec{U_{\chi_{1}}}-2\vec{U}\left(t\right)\right)$$
  where *bb*_1_ and *bb*_2_ are constant coefficients in the range of (0, 1] for change adjustment exploration capability; *cc*_1_ and *cc*_2_ are uniform random coefficients.

In this paper, the ACGWO-BP algorithm is developed for the parameter search of BP algorithm by combining the improvement points, and its procedure is shown in Table 4. The BP network fitting error RMSE is used as the fitness function for the population search within the designated number of iterations, subject to the initialization settings of the BP network and the ACGWO algorithm. At the conclusion of the ACGWO search, the optimal solution is utilized as the weight and threshold of the BP network for training purposes. The network training results are then output to form the fitting model, and the test samples are used to validate the fitting model.

#### 3.2.2. ACGWO-BP-NSGA-II Optimization Framework

**Figure 10:**
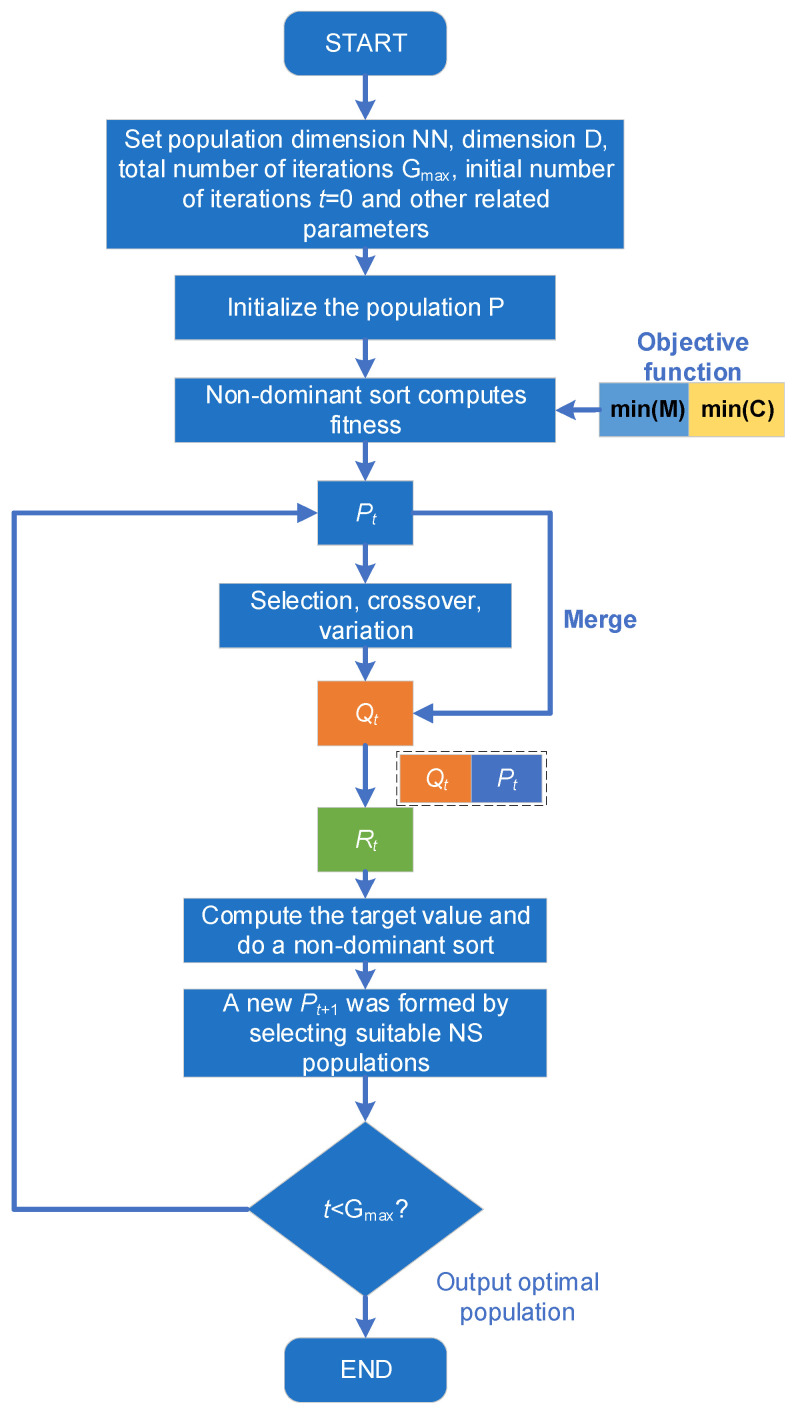
ACGWO-BP-NSGA-II design block diagram.

Considering that the two optimization objectives are interrelated and need to be balanced and coordinated to ensure that each objective achieves optimal results for practical engineering needs. The NSGA-II genetic algorithm [39], a non-dominated sorting genetic algorithm, is selected in this paper for multi-objective optimization of multi-stage size structures. The NSGA-II genetic algorithm performs non-dominated sorting on the initial population, and then similar to the traditional genetic algorithm, performs screening, cross, and mutation to obtain new populations, followed by merging the sub-populations with the parent population, performing non-dominated sorting to obtain the frontier of non-dominated solutions, and using the crowding distance as a secondary criterion to maintain the diversity of solutions. The non-dominated sort in this algorithm is fast and can optimize the Pareto domination time complexity from O(NN^3^) down to O(NN^2^), and the proposed crowding distance can globally capture the distribution of solutions and ensure the spatial uniformity of solutions. Finally, an improved non-dominated ranking genetic algorithm (ACGWO-BP-NSGA-II) optimization design framework is built in this paper as shown in Figure 10.

First, initialize the relevant parameters such as population number NN, dimension D, and maximum number of iterations *G*_max_. Initialize population P simultaneously. Then, produce the initial population *P*_t_, calculate the individual fitness of population *P_t_*, and subsequently conduct an iterative session. Through crossover, mutation, and selection, a new offspring population *Q_t_* is produced, and *Q_t_* is spliced with *P_t_* to form a new population *R*_t_. In the iteration process, the target value M and C are calculated and non-dominated ranking is performed for the new population *R_t_*, and the population members of *R*_t_ are sorted to different frontiers according to non-dominated ranking. Subsequently, the appropriate NS individuals are selected as the new population *P_t_*_+1_ until the number of iterations *t* is completed. Finally, the population with the result of the last iteration is selected as the best population of individuals output.

### 3.3. Results Validation and Analysis

**Figure 11:**
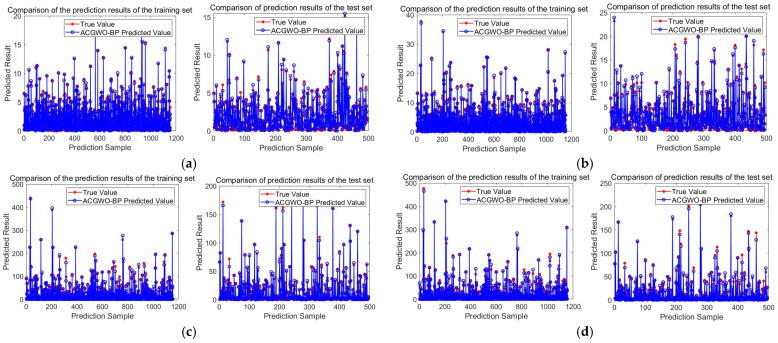
The effect of ACGWP-BP fitting under each disturbance: (**a**) Under interference A; (**b**) Under interference B (**c**) Under interference C; (**d**) Under interference D.

To establish the *F_i_* fitting function, 80% of the data were selected at random as the data set, and the remaining data were fitted for validation. The initialization settings for the parameter optimization of the ACGWO algorithm were 60 for the population individuals and 50 for the maximum number of iterations; the number of layers of the BP network was set to 5, with three hidden layers, one input layer, and one output layer; the number of network iterations was 100; and the convergence threshold was 0.00001. The fitting effect of *F*_1_–*F*_4_ is shown in Figure 11.

As can be seen from Figure 11, the ACGWO-BP network training is not over-fitted, and the fit is good. Since disturbance 3 and disturbance 4 have a large impact on the TMR sensor, the overall MC value is high, so the *MAE* will be higher. Looking over the original fitting data, it can be found that under the influence of small disturbance currents, the *MAE* of the *F*_3_ fitting is about 0.53, and the *MAE* of the *F*_4_ fitting is about 0.45, which indicates that the proposed algorithm can obtain a good fitting effect even for samples with a large span of output data.

Table 5 compares the proposed ACGWO-BP method to commonly used fitting methods, revealing that the proposed ACGWO-BP method in this paper has a higher level of accuracy for processing nonlinear fitting problems.

After obtaining the *F_i_* fitting formula, the multi-objective optimization problem to be solved is changed to Equation (20).

(20) $$\begin{array}{l} minM=z_{1}F_{1}(X){+z}_{2}F_{2}(X){+z}_{3}F_{3}(X){+z}_{4}F_{4}\left(X\right)\\ minC=p+th_{1}+th_{2}\\ st.\left\{\begin{array}{c} p\in \left[6,15\right]\\ th_{1}\in \left[1,10\right]\\ th_{2}\in \left[1,10\right] \end{array}\right. \end{array}$$

*z*_1_–*z*_4_ represent the influence weights of *I′_A_–I′_D_*, respectively, which are determined based on the information of the relative size of *MC* obtained from each angle and fluctuations, and based on the sample *MC* values, according to the weighted average method and the entropy weight method, it is determined that *z*_1_ = 0.057, *z*_2_ = 0.155, *z*_3_ = 0.390, *z*_4_ = 0.398.

**Figure 12:**
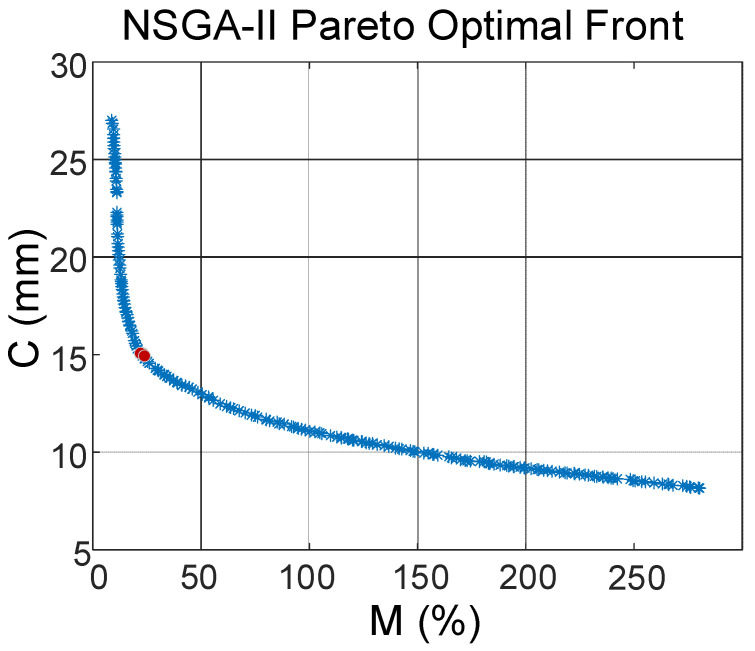
Pareto Frontier Diagram.

The magnetic field measurement near a substation arrester leakage current measurement in southwest China is subjected to electromagnetic interference of about 1 μT. During the simulation experiment of this paper, when *I′* = 1 A, the magnetic field interference near the measurement point is consistent with the field order of magnitude, and at the same time, as *I* increases, the external anti-magnetic interference capability of the set size is relatively consistent, the obtained optimal size can still be applied. The NSGA-II algorithm is used for size finding, setting *I′* = 1 A, the initial population is 200, the elite ratio is 0.2, and the evolutionary generation is 50, to obtain the Pareto frontier shown in Figure 12.

The Pareto frontier is a series of compromise solutions in line with the multi-objective optimization range, as shown by the blue curve in Figure 12. To balance the relationship between size and interference resistance, the optimal set of solutions corresponding to the turning point of the decreasing slope in the boundary diagram is selected as the final solution of this optimization and marked with red dots. The results of the optimization search at the red dots marked in the figure are shown in Table 6.

From Table 6, the combined measurement error and the trend of magnetic field under each size of magnetic ring structure calculated by the ACGWO-BP-NSGA-II algorithm are consistent with the general trend of the finite element simulation results, which verifies the effectiveness of the structure optimization design proposed in this paper. The optimized set of three-stage ring sizes shows an average decrease in 105% in the measurement error compared with the single ring structure of the same thickness, and when the total thickness of the three-stage ring is less than 25 mm, the reduction in the total thickness brings a rapid increase in the measurement error. Further, the magnitude of the magnetic field sensitivity of the deployment point for each level of thickness design was calculated, as shown in Table 7, and the designed multi-stage ring structure still has good front-end magnetic field amplification.

Regarding comprehensive magnetic field measurement sensitivity and anti-magnetic anti-interference performance, and taking into account the size of the sensor, the final determination of the 3-stage magnetic ring size inner diameter *r*_1_ = 10 mm, *p* = 6 mm, *th*_1_ = 1 mm, *th*_2_ = 8.3 mm, *h* = 10 mm, *d* = 8 mm.

## 4. Experimental Verification

**Figure 13:**
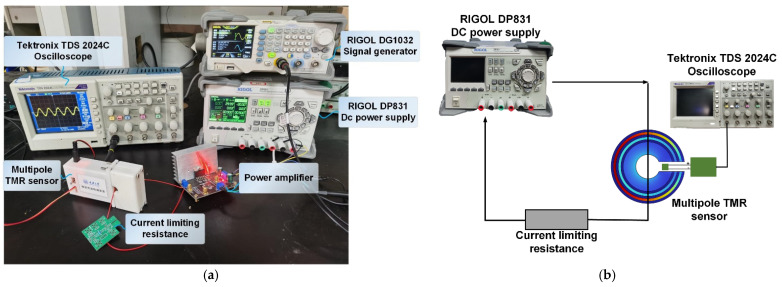
Experimental test platform: (**a**) AC characteristics experimental platform; (**b**) DC characteristics experiment platform.

In this paper, an AC/DC measurement experimental platform is built as shown in Figure 13. The output of the signal source forms a complete loop with the current-limiting load and passes through the magnetic ring of the TMR sensor in order to achieve the measurement of the target current. The TMR sensor hardware circuit involved in the experimental platform consists of a front-end sensing module, a signal amplification and acquisition module, a zeroing module, and a power supply module. The front-end sensing module is selected from the TMR2905 chip produced by (Jiangsu Dovetail Technology Co., Ltd., Suzhou, China), which has high sensitivity and low noise. The signal amplification and acquisition module consists of the primary amplification composed of AD8429 (Adeno Semiconductor Technology (Shanghai) Co., Ltd., Shanghai, China) and the second amplification circuit composed of AD8012 (Adeno Semiconductor Technology (Shanghai) Co., Ltd., Shanghai, China). The zeroing module introduces the secondary amplification and constitutes a subtractive circuit to realize hardware zeroing. The power supply module is responsible for supplying energy to each chip. In the actual design process, the power supply line is designed in layers with the signal line, and a decoupling circuit is introduced to suppress the noise interference introduced by the power supply.

**Figure 14:**
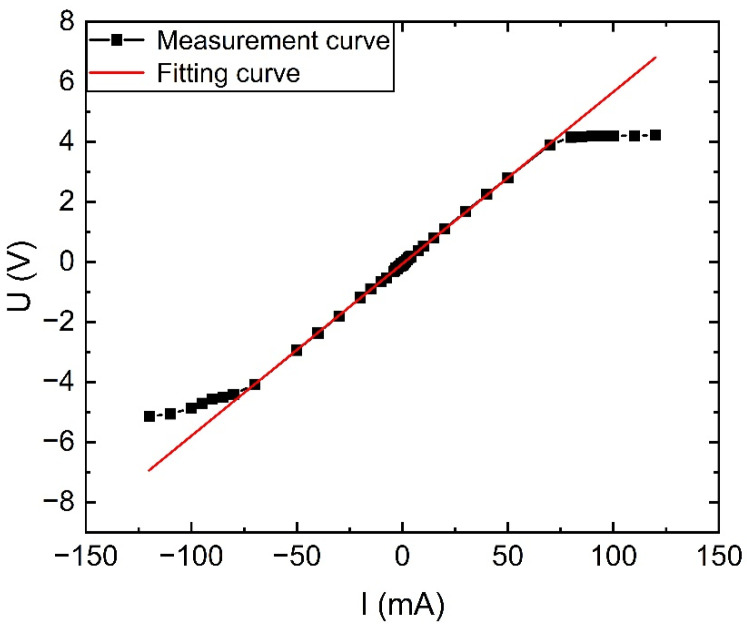
Sensor range and linearity.

**Figure 15:**
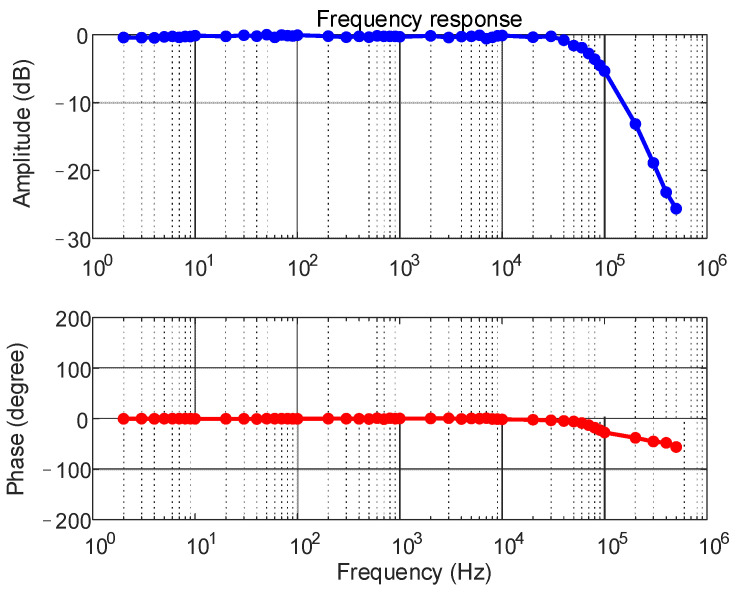
Sensor frequency response characteristics.

The TMR sensor designed in this paper was tested for range and amplitude-frequency characteristics, and the measurement results were obtained as shown in Figure 14 to Figure 15. From the figures, it can be seen that the TMR sensor designed in this paper can respond to variable DC between ±60 mA with fitting error less than 1% and has 80 kHz measurement bandwidth.

To verify the stability enhancement of the proposed new multi-stage magnetic ring structure for small current measurement, this paper focuses on the AC/DC measurement experiments from 0–1 mA and analyzes and illustrates the minimum AC value or DC magnitude that can be measured. The new multi-stage TMR sensor is experimentally re-compared with the TMR sensor under single magnetic ring structure under weak and strong electromagnetic disturbances.

### 4.1. AC Characteristics Experiment

#### 4.1.1. No External Interference Situation

**Figure 16:**
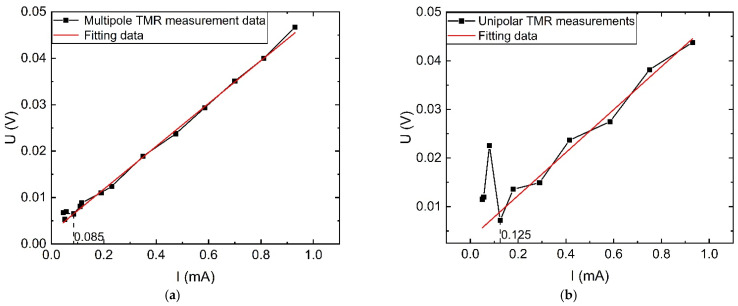
Comparison of TMR sensor input and output AC characteristic curves under no interference: (**a**) Under multi-stage magnetic ring structure; (**b**) Under monostage magnetic ring structure.

In the AC characteristic experimental platform, the signal generator output is passed through the power amplifier to form a complete circuit with the current-limiting load and through the TMR sensor magnetic ring to achieve the measurement of the target current. The signal generator is adjusted to generate current outputs of different amplitudes with a frequency of 50 Hz, and the original side currents and the corresponding output voltages of the TMR sensors are recorded separately to obtain the input–output characteristic curves of multi-stage TMR and single-stage TMR as shown in Figure 16.

When the sensor measurement range is set to 0–1 mA current, the sensor sensitivity is 46.1 mV/mA, as can be seen from Figure 16a. Additionally, the minimum measurement value of the new multi-stage TMR sensor can reach 85 μA, and the measurement error at this current value is 0.3%, while the single-stage TMR in measuring the current value of 85 μA has produced a huge deviation with an error of 200%. The nonlinear error of the multi-stage TMR sensor at 85 μA–1 mA is 2.2% according to Equation (21), and the nonlinear error of the single-stage TMR sensor at 125 μA–1 mA is 20.5%. The above analysis shows that the new multi-stage magnetic ring structure can effectively reduce the minimum measurement value of the weak sensor and improve the stability of the current measurement at the microampere level.

(21) $$\delta =\frac{\Delta Y_{max}}{Y_{FS}}\times 100\%$$

In the Equation (13), Δ*Y_max_* is the maximum deviation of the measured value from the fitted curve, and *Y_FS_* is the range.

#### 4.1.2. External Magnetic Interference Situation

**Figure 17:**
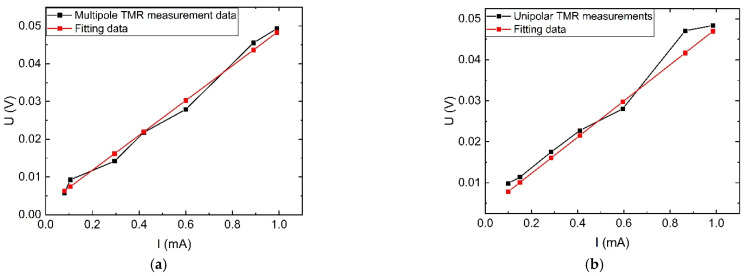
Comparison of the input and output AC characteristics of TMR sensors under interference: (**a**) Under multi-stage magnetic ring structure; (**b**) Under monostage magnetic ring structure.

In the experimental process, a constant DC source was used to output 15 A current, placed near the target measurement wire to simulate a strong magnetic field interference application scenario, the measured target current magnitude was gradually changed according to the steps in Section 4.1.1, and the measured voltage output of the sensor was compared and analyzed with the value of the sensor fitting curve in Section 4.1.1. From Figure 17, it can be seen that at 0–1 mA, the nonlinear error of the new TMR sensor increases to 6%, and the nonlinear error of the monostage TMR sensor reaches 30%. The experimental results show that for AC measurement, the designed multi-stage TMR sensor can still maintain high accuracy for small current measurements under external interference.

### 4.2. DC Characteristics Experiment

**Figure 18:**
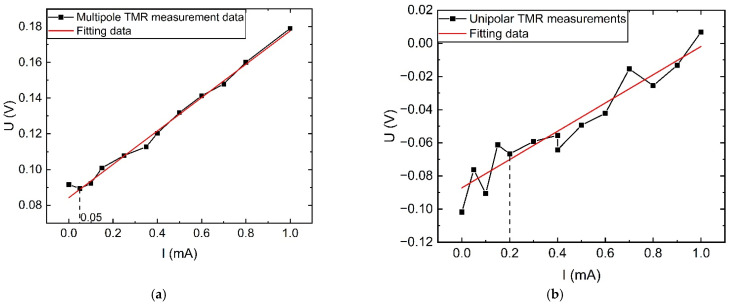
Comparison of TMR sensor input and output DC characteristic curves under no interference: (**a**) Under multi-stage magnetic ring structure; (**b**) Under monostage magnetic ring structure.

In the DC stage shown in Figure 13b, the designed sensor is measured for 0–1 mA DC, similar to the steps in Section 4.1, to obtain the TMR sensor input–output characteristic curve as shown in Figure 18.

As can be seen in Figure 18, the input–output characteristic curve of the multi-stage TMR sensor has a better linear fit, with a nonlinear error of less than 4.5% and a minimum measurement down to 50 μA. In the single-loop structure, the nonlinear error is 17% and the minimum measurable value is 200 μA. In DC small-current measurements, the multi-stage TMR sensors also highlight their advantages in DC small current measurements.

**Figure 19:**
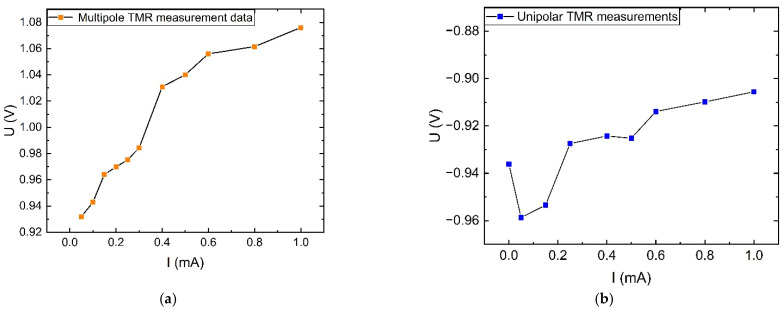
Comparison of the input and output DC characteristics of TMR sensors under interference: (**a**) Under multi-stage magnetic ring structure; (**b**) Under monostage magnetic ring structure.

For DC characteristic investigations, strong interference currents were again positioned close to the measurement leads. Without other shielding measures, the strong interfering magnetic field will introduce more magnetic noise and cause larger measurement errors in the microampere level DC measurements, as shown in Figure 19. However, comparing the values of the curves measured by the multi-stage TMR sensor to those of the single-stage TMR sensor reveals that the multi-stage TMR sensor can still maintain a good response trend under strong interfering magnetic fields.

### 4.3. Performance Comparison Analysis

Table 8 gives a comparison of the latest performance of the sensors designed in this paper with other magnetoresistive sensor technologies based on non-contact measurement techniques and oriented to weak current measurements for a more in-depth discussion of the work in this paper.

Due to the fact that the design of different sensors is dependent on the process of the front-end magnetic sensor chip and the selection and configuration of the electronics, it is difficult to make a direct comparison; however, a comparative analysis can be conducted based on the performance parameters that the sensors ultimately embody. As shown in Table 8, TMR-based sensing technology has a higher measurement sensitivity than GMR-based sensing technology. The multi-stage TMR leakage current sensor designed in this paper has certain advantages in terms of measurement sensitivity, minimum current measurement value, and bandwidth, and it has increased potential for applications involving current measurement under complex operating conditions. Despite the fact that the measurement range of the sensor designed in this paper is restricted to a small range in order to improve the performance advantage in small current measurement, it can be easily extended to large current measurement with the help of an alternative concept in order to achieve superior performance in large current measurement.

However, due to the multi-stage magnetic ring design, there is no significant reduction in the cost of the sensor designed in this paper without commercialization; and when it is used in outdoor online monitoring for a long time, the influence of its hysteresis performance and temperature performance on the measurement will not be ignored, and further in-depth research is needed.

## 5. Conclusions

In this paper, a new multi-stage TMR sensor structure is presented, the front-end measurement characteristics are analyzed and summarized, and the multi-stage TMR sensor size optimization and field experimental analysis are conducted. From the analysis of front-end measurement characteristics, it can be deduced that the front-end magnetic field measurement sensitivity is primarily dependent on the thickness of the inner-stage polymagnetic layer, and that the multi-stage structure has better front-end magnetic field amplification than the single-ring structure as the thickness of the inner-stage polymagnetic layer varies. The simulation results demonstrate that the interference immunity of the multi-stage ring structure is substantially greater than that of the single-stage ring structure, with the open air gap width *d* and the size matching of each stage having the greatest impact on the characteristics. The ACGWO-BP-NSGA-II optimized sizing framework determines the inner diameter of the 3-stage ring size with *r*_1_ = 10 mm, *p* = 6 mm, *th*_1_ = 1 mm, *th*_2_ = 8.3 mm, *h* = 10 mm, and *d* = 8 mm. The proposed multi-stage magnetic ring structure TMR current sensor reduces measurement error by 105% compared to a single magnetic ring TMR current sensor of the same volume.

From the field experimental results, it can be seen that the multi-stage TMR sensor has better stability than the traditional single-loop open-loop TMR sensor in microamp level current measurement, the non-linear error of AC and DC measurement is reduced by 13.5%, the minimum value of AC measurement is reduced from 125 μA to 85 μA, and the minimum value of DC measurement is reduced from 200 μA to 50 μA. The experimental results confirm the measurement stability of the proposed structure under external disturbances and provide ideas and directions for the engineering application of TMR AC/DC detection technology.

However, the hysteresis characteristics of the multi-stage ring structure have an impact on the measurement accuracy of the sensor in long-term operation, and the TMR sensor measurement is influenced by the bias voltage. In the subsequent work, the hysteresis characteristics of the multi-stage ring will be tested and studied, and a relevant demagnetization scheme will be designed to further improve the accuracy of the measurement at weak currents. Meanwhile, considering the demand of online monitoring, the designed multi-stage TMR sensor bias voltage elimination circuit and temperature compensation circuit are further enhanced, and the addition of external shielding shell will be considered to adapt to the influence of unknown electromagnetic interference from multiple sources and improve the stability and measurement accuracy of the designed sensor engineering applications.

## Figures and Tables

**Table 1:** Material settings.

Material Name	Relative Permeability
Permalloy	50,000
Aluminum	1
Copper	1

**Table 2:** Model initialization values.

Model Label Name	Size (mm)
*r*	2
*r*_1_	10
*h*	10
*d*	8
*p*	10
*th*_1_–*th_N_*_−1_	2

**Table 3:** Setting of the thickness of each stage of the multi-stage ring structure.

Level *N*	Size Setting
1	*p* = 10 mm
2	*p* = 5 mm, *th*_1_ = 15 mm
3	*p* = 3 mm, *th*_1_ = 3 mm, *th*_2_ = 4 mm
4	*p* = 2.5 mm, *th*_1_ = 2.5 mm, *th*_2_ = 2.5 mm, *th*_3_ = 2.5 mm
5	*p* = 2 mm, *th*_1_ = 2 mm, *th*_2_ = 2 mm, *th*_3_ = 2 mm, *th*_4_ = 2 mm
6	*p* = 1.6 mm, *th*_1_ = 1.6 mm, *th*_2_ = 1.6 mm, *th*_3_ = 1.6 mm, *th*_4_ = 1.6 mm, *th*_5_ = 2 mm

**Table 4:** ACGWO-BP training process.

Training Process
■ Initialize BP network, determine the number of implied layers *kk*; number of nodes *hh_k_*; loss function *MAE*; output node excitation function ReLU. ■ Initialize ACGWO algorithm parameters: number of gray wolf populations NM; the maximum number of iterations *t*_max_; number of iterations l; optimal search dimension dim, dimension upper session ub, dimension lower bound ul; location update factors *bb*_1_ and *bb*_2_; input adaptation function fun; initialize wolf population location according to Equation (17). ■ Train the gray wolf algorithm in each iteration: For each wolf pack, ♦ Update the optimal solution (position of the head wolf *χ*_1_) according to Equation (19); Updated $\vec{a}$, $\vec{AA}$, $\vec{CC}$ Calculate fun function values; Update the location of *χ*_1_, *χ*_2_, *χ*_3_; Number of update iterations; ■ End of iteration, output BP neural network optimization weights and thresholds for fitting training. ■ Model is built successfully for verification.

**Table 5:** Comparison of the fitting effect of each algorithm.

**Interference A**				
**Algorithm Name**	***R* ^2^_train_**	***MAE*_train_**	***R* ^2^_test_**	***MAE*_test_**
Random forest	0.94	0.38	0.87	0.6
GRNN	0.98	0.2	0.95	0.35
Bipolar rational fractions	0.84	0.69	0.82	0.7
BP	0.89	0.54	0.87	0.58
ACGWO-BP	0.99	0.10	0.97	0.12
**Interference B**				
**Algorithm name**	***R* ^2^_train_**	***MAE*_train_**	***R* ^2^_test_**	***MAE*_test_**
Random forest	0.88	0.8	0.78	1.16
GRNN	0.99	0.25	0.77	1
Bipolar rational fractions	0.74	1.5	0.707	1.43
BP	0.8	1.28	0.72	1.41
ACGWO-BP	0.97	0.14	0.92	0.17
**Interference C**				
**Algorithm name**	***R* ^2^_train_**	***MAE_t_*_rain_**	***R* ^2^_test_**	***MAE*_test_**
Random forest	0.97	3.25	0.88	4.42
GRNN	0.98	1.01	0.96	2.81
Bipolar rational fractions	0.95	4.46	0.94	4.62
BP	0.96	3.50	0.92	3.80
ACGWO-BP	0.99	1.48	0.96	1.69
**Interference D**				
**Algorithm name**	***R* ^2^_train_**	***MAE*_train_**	***R* ^2^_test_**	***MAE*_test_**
Random forest	0.93	3.31	0.88	4.06
GRNN	0.99	0.7	0.93	3.02
Bipolar rational fractions	0.96	4.50	0.95	4.40
BP	0.98	2.8	0.97	3.02
ACGWO-BP	0.99	1.25	0.98	1.24

**Table 6:** The combined error of magnetic field measurement at each stage thickness.

*p* (mm)	*th*_1_ (mm)	*th*_2_ (mm)	M (%) (NSGA-II)	M (%) (Multi-Stage Magnetic Ring)	M (%) (Single Magnetic Ring)
6.6	1	8.5	18.32	16.7	104
6	1	8.7	19.23	16.6	122
6	1	8.4	20.08	19.1	105
6	1	8.3	20.69	19.0	138
6	1	8	22.03	19.8	142
6	1	7.9	22.88	20.0	138
6	1	7.6	25.19	23.6	139
6	1	7.5	26.02	24.5	120

**Table 7:** Sensitivity of magnetic field measurement at each stage thickness.

*p* (mm)	*th*_1_ (mm)	*th*_2_ (mm)	A (Multi-Stage Magnetic Ring)	A (Single Magnetic Ring)
We a6.6	1	8.5	15.0	15.2
6	1	8.7	15.1	15.3
6	1	8.4	15.0	15.2
6	1	8.3	15.0	15.3
6	1	8	15.0	15.3
6	1	7.9	15.0	15.3
6	1	7.6	15.0	15.2
6	1	7.5	15.0	15.3

**Table 8:** The latest performance comparison of magnetoresistive current sensor technology.

Parameter	This Work	[32]	[31]	[30]	[40]
Sensor technology	TMR	TMR	TMR	GMR	GMR
Sensor setup sensitivity	57.30 V/A	10.52 V/A	10 V/A	0.0392 V/A	0.2319 V/A
Measurement range	±60 mA	±300 mA	±200 mA	±200 mA	±300 mA
Detection limit	AC: 85 μA DC: 50 μA	AC: 280 μA	/	10 mA	AC: 100 to 300 μA DC: 100 μA
Bandwidth	80 kHz	10 kHz	/	1 MHz	50 kHz

## Data Availability

The data that supports the findings of this study is available from the corresponding author upon reasonable request.

## Nomenclature

The following abbreviations and symbols are mainly used in this manuscript:

TMR	Tunneling magnetoresistive
AMR	Anisotropic magnetoresistive
GMR	Giant magnetoresistive
A	Magnetic field measurement sensitivity
MC	Relative error of magnetic field measurement
M	Combined error of magnetic field measurement
C	The total thickness of the magnetic ring
F	Fitting the mapping
ACGWO	Adaptive Gray Wolf Algorithm
ACGWO-BP-NSGA-II	The improved non-dominated ranking genetic algorithm
R^2^	The goodness-of-fit
MAE	The mean absolute error
B	Magnetic induction strength magnitude
N	Number of stages of multi-stage magnetic ring
h	Height of multi-stage magnetic ring
p	Thickness of inner stage polymagnetic layer
r	Radius of lead wire
r_1_	Inner radius of multi-stage magnetic ring
th_i_	Thickness of attenuation layer at each level
d	Width of air gap of magnetic ring opening
I′_i_ (i = A–D)	Interference current in four directions
δ	Non-linear error
τ	The electromagnetic propagation coefficient
μ	Permeability
α	Dielectric constant
β	Phase constant
σ	Electrical conductivity
